# Correlation of Red Cell Distribution Width (RDW) With Weight Loss and Grade of Mucositis in Patients Who Underwent Head and Neck Radiotherapy: A Retrospective Study

**DOI:** 10.7759/cureus.88991

**Published:** 2025-07-29

**Authors:** Sri Vani Chandana, Manjunath G. N., S. M. Azeem Mohiyuddin, Kalyani Raju

**Affiliations:** 1 Otorhinolaryngology-Head and Neck Surgery, Sri Devaraj Urs Medical College, Kolar, IND; 2 Radiation Oncology, RL Jalappa Hospital and Research Centre, Kolar, IND; 3 Pathology, Sri Devaraj Urs Medical College, Kolar, IND

**Keywords:** head and neck cancer, inflammation, mucositis, prognostic biomarker, radiotherapy, radiotherapy induced complications, red cell distribution width (rdw), weight loss

## Abstract

Background: Red cell distribution width (RDW) is an emerging biomarker associated with systemic inflammation, nutritional status, and prognosis in various cancers. However, its correlation with treatment-related toxicities such as mucositis and weight loss in head and neck cancer patients undergoing radiotherapy remains underexplored.

Objective: This study aimed to evaluate the correlation between RDW (pre-treatment, post-treatment, and delta) with weight loss and mucositis severity in patients who received head and neck radiotherapy, with or without concurrent chemotherapy.

Methods: A retrospective analysis was conducted on 165 patients treated between 2023 and 2024 at Sri Devaraj Urs Medical College, Kolar. Ethics approval was obtained from the Institutional Ethics Committee, and informed consent was waived owing to the retrospective nature of the study. Patients with recurrent disease, inflammatory or autoimmune disorders, or second malignancies were excluded. Weekly data on weight, mucositis grade, and hematological parameters, including RDW, were extracted from case records. Statistical analysis was performed using IBM SPSS Statistics for Windows, Version 26 (Released 2020; IBM Corp., Armonk, New York, United States), and Spearman correlation was used to assess associations.

Results: RDW delta showed a significant positive correlation with weight loss from week 2 to week 7, with the strongest association in week 6 (r = 0.253, p = 0.002). Logistic regression confirmed RDW delta was a statistically significant marker associated with >10% weight loss (odds ratio (OR) = 1.13, p = 0.032). In contrast, RDW showed weak and inconsistent correlations with mucositis grade, achieving significance only in week 2 (r = 0.225, p = 0.005).

Conclusion: RDW delta is a promising, cost-effective biomarker associated with weight loss during head and neck radiotherapy. However, its role as a marker for mucositis severity appears limited.

## Introduction

Red cell distribution width (RDW) is a routinely available hematological parameter that reflects the heterogeneity in the size of circulating erythrocytes, traditionally used to aid in the diagnosis of various forms of anemia. In recent years, RDW has garnered significant attention as a potential biomarker of inflammation, nutritional status, and clinical outcomes in diverse malignancies [1]. Its clinical appeal lies in its cost-effectiveness, universal availability, and ease of integration into standard diagnostic workflows.

The prognostic significance of RDW has been demonstrated across multiple solid tumors. In a large retrospective cohort of patients with endometrial carcinoma, elevated preoperative RDW was independently associated with advanced disease stage, pelvic lymph node metastasis, and significantly shorter disease-free and overall survival [1]. Similarly, a systematic review and meta-analysis by Nocini et al. (2023) found that RDW was a robust predictor of survival in patients with laryngeal cancer, correlating strongly with both disease-free and overall survival rates [2]. In colorectal cancer, increased RDW has been linked not only to poorer nutritional and immune status, but also to more advanced tumor characteristics and reduced survival outcomes [3].

A growing body of evidence also supports the prognostic value of RDW in pancreatic and gastric cancers. In pancreatic cancer, patients with elevated preoperative RDW were found to have worse immune-nutritional profiles and significantly poorer postoperative survival, even after adjusting for confounders such as age and tumor stage [4]. Similarly, in gastric cancer, high RDW values were significantly associated with deeper tumor invasion, lymph node metastasis, and shorter survival durations [5].

Despite its consistent association with survival outcomes, the utility of RDW as a predictive biomarker for treatment-related toxicities in cancer patients remains underexplored. In particular, few studies have examined whether changes in RDW (RDW delta) during treatment may serve as an early marker for complications such as weight loss, mucositis, or the need for nutritional support. These outcomes are especially relevant in patients with head and neck cancers undergoing radiotherapy, where inflammation, tissue damage, and catabolic stress are common and often lead to rapid nutritional decline.

Therefore, this study aims to address this gap by evaluating both static RDW measures and dynamic RDW delta as predictors of adverse treatment outcomes in this patient population. By investigating their correlation with mucositis severity, weight loss, and nasogastric tube (NGT) insertion, we aim to assess the potential of RDW to serve as a simple, non-invasive biomarker for early risk stratification and nutritional intervention.

## Materials and methods

This retrospective observational study was conducted in the Department of Otorhinolaryngology (ENT) at Sri Devaraj Urs Medical College, Kolar, India. Ethics approval was obtained from the Institutional Ethics Committee (Ref. No. SDUAHER/KLR/R&D/CEC/S/PG/62/2024-25) and informed consent was waived owing to the retrospective nature of the study. The study included patients diagnosed with head and neck cancer who underwent either radical or adjuvant radiotherapy, with or without concurrent chemotherapy. The data collection spanned from 2023 to 2024, allowing for a sufficiently large and diverse cohort representative of real-world clinical practice. This extended study period enabled the inclusion of varying treatment regimens and ensured adequate longitudinal follow-up for weekly clinical assessments.

Inclusion and exclusion criteria

To ensure methodological consistency and minimize confounding, only treatment-naïve patients were included. Specifically, patients with a history of prior disease recurrence, autoimmune conditions, chronic inflammatory disorders, or second primary malignancies were excluded. These exclusion criteria were deliberately chosen to reduce the influence of systemic inflammatory states or comorbidities on hematological parameters, particularly RDW, which served as the study’s primary variable of interest.

Ethical considerations

Prior to data collection, ethical approval was obtained from the Central Ethics Committee, Sri Devaraj Urs Academy of Higher Education and Research, Kolar. Given the retrospective design of the study, informed consent was waived. Nevertheless, all patient records were handled with strict confidentiality, and data were anonymized at the point of extraction to protect patient identity and uphold ethical standards in research.

Data collection and variables

Patient information was retrospectively extracted using a standardized data collection form. Demographic variables, including age and sex, were recorded, along with detailed clinical data such as tumor location, staging, chemotherapy status, total radiation dose, number of fractions, and treatment duration. Each treatment week was defined as a cycle of five consecutive daily radiation fractions.

Weekly clinical assessments were focused on two primary outcomes: mucositis grade and body weight. Mucositis was graded using the Radiation Therapy Oncology Group (RTOG) criteria from week 2 through week 7 of treatment. Similarly, weight loss was documented weekly and expressed as a percentage relative to baseline body weight.

In terms of hematological evaluation, RDW values were recorded at two time points: prior to the initiation of radiotherapy (pre-treatment RDW) and within one week after the completion of radiotherapy (post-treatment RDW). The difference between the post-treatment value and the initial pre-treatment value (RDW delta) was also calculated and included in the analysis.

Importantly, week 8 data were excluded from all statistical analyses due to an insufficient sample size (n=5). This decision was based on the fact that only five patients, all from the chemoradiotherapy subgroup, had a scheduled follow-up during that week. Such a limited sample was deemed insufficient for meaningful statistical interpretation and would have introduced bias in subgroup comparisons and regression models.

Sample size determination

The sample size was determined using prevalence estimates and statistical parameters drawn from prior literature. Specifically, the methodology referenced the study by Ge et al. [6]. Using local epidemiological data, the estimated cancer prevalence in the Kolar region was approximately 23.65%, with a complementary proportion (Q) of 76.35%. Applying an allowable error margin of 10%, the calculated minimum required sample size was 165 patients. This number was adequate to support the study’s correlation and regression analyses, while also allowing for stratification by chemotherapy status.

Statistical analysis

Following data extraction, all variables were entered into Microsoft Excel (Microsoft® Corp., Redmond, WA, USA) for initial organization and validation. Statistical analysis was conducted using IBM SPSS Statistics for Windows, Version 26 (Released 2020; IBM Corp., Armonk, New York, United States). Descriptive statistics were employed to summarize the characteristics of the study population. Categorical variables such as gender and chemotherapy status were reported as frequencies and percentages, while continuous variables, including age, weight, and RDW values, were expressed as means with standard deviations.

For inferential analysis, Spearman's rank correlation coefficient was used to examine associations between RDW metrics (pre-treatment, post-treatment, and delta) and clinical outcomes, specifically weekly weight loss and mucositis grade. Spearman's method was chosen due to the non-parametric nature of the data and the ordinal scale of mucositis grading. Correlation coefficients were computed for each week of treatment from week 2 through week 7, with statistically significant p-values (<0.05) identified using asterisk notation in summary tables.

To further assess the correlative value of RDW, binary logistic regression was performed with two defined endpoints: (1) severe weight loss, categorized as >5% and >10% loss from baseline; and (2) severe mucositis, defined as WHO grade ≥3. Separate regression models were constructed using each RDW measure as an independent variable. Odds ratios (ORs) with 95% confidence intervals (CIs) were calculated to quantify the strength of association. The discriminative ability of each model was evaluated using the area under the receiver operating characteristic (ROC) curve (AUC).

Weight loss was clearly defined as the percentage reduction from baseline weight at each weekly visit, with severe weight loss defined a priori as >5% and >10% reduction from baseline body weight. In correlation analyses, we used the continuous percentage change each week; in logistic regression, we examined the endpoint of >10% weight loss at treatment completion. In addition to the overall analysis, a weekly logistic regression was performed to evaluate whether RDW parameters could correlate with adverse outcomes on a week-by-week basis. This granular approach provided insight into the temporal evolution of RDW's correlative utility during the course of radiotherapy. A p-value of less than 0.05 was considered indicative of statistical significance for all tests.

## Results

A total of 165 case records of patients with head and neck cancer were screened between 2023 and 2024. Following the application of exclusion criteria, which included incomplete data, recurrent disease, inflammatory or autoimmune disorders, and second malignancies, 12 patients were excluded. Consequently, the final study cohort consisted of 153 patients. Among these, 73 patients received concurrent chemoradiotherapy, while 80 underwent radiotherapy alone.

The baseline characteristics of the cohort revealed a mean age of 59 years (±13), with a median of 60 years (Table 1). The study included 83 female participants and 70 male participants (54.2% and 45.8%, respectively). Carcinoma of the buccal mucosa (CaBM) was the most prevalent diagnosis (n = 64, 41.8%), followed by carcinoma of the tongue (n = 12, 7.8%). The most common stages were T3N0M0 (n = 31, 20.3%), T2N0M0 (n = 21, 13.7%), and T4N0M0 (n = 14, 9.2%). Patients received a mean radiation dose of 61.7 Gy (±3.0) delivered over an average of 31 fractions (±2). The mean pre-treatment body weight was 53.6 kg (±12.9), and the mean pre-treatment RDW was 14.5% (±2.3).

**Table 1:** Baseline Demographics and Clinical Characteristics SD: standard deviation; BM: buccal mucosa; Ca: carcinoma; GBS: gingivobuccal sulcus

Variable	Subcategory	Raw Values
Age (years)	Mean ± SD	59 ± 13
	Median (IQR)	60.0 (49.0-69.0)
	Range	30-94
Sex	Female (F)	83 (54.2%)
	Male (M)	70 (45.8%)
Diagnosis (top 5)	Ca.BM	64 (41.8%)
	Ca.TONGUE	12 (7.8%)
	Ca.UPPERGBS	9 (5.9%)
	Ca.LOWERGBS	8 (5.2%)
	Ca.HARDPALATE	7 (4.6%)
Stage (top 5)	T3N0M0	31 (20.3%)
	T2N0M0	21 (13.7%)
	T4N0M0	14 (9.2%)
	T2N2M0	10 (6.5%)
	T4N2M0	10 (6.5%)
Chemotherapy	No (N)	80 (52.3%)
	Yes (Y)	73 (47.7%)
Radiation dose (Gy)	Mean ± SD	61.7 ± 3.0
	Median (IQR)	60.0 (60.0-64.0)
Dose per fraction (Gy)	Mean ± SD	2.0 ± 0.1
	Median	2.0
Number of fractions	Mean ± SD	31 ± 2
	Median	31 (30-33)
Pre-treatment weight (kg)	Mean ± SD	53.6 ± 12.9
	Median (IQR)	54.0 (45.0-60.0)
RDW pre-treatment (%)	Mean ± SD	14.5 ± 2.3
	Median (IQR)	13.9 (12.9-15.0)

Follow-up and data completeness

Weekly data were collected for each participant from weeks 1 to 8. However, due to attrition and limited follow-up compliance, week 8 data were excluded from all analyses, as only five patients completed assessments during that period. Notably, there were no dropouts until week 4, when two deaths occurred. By week 6, three patients had either died or were lost to follow-up. Attrition increased significantly in week 7, with 10 patients withdrawing or being lost for unspecified reasons.

Correlations between RDW and mucositis grade

To examine the relationship between RDW and mucositis, Spearman correlation coefficients were calculated for both pre-treatment and post-treatment RDW values across weeks 2 to 7 (Table 2). The most noteworthy finding was observed in week 2, where post-treatment RDW showed a significant positive correlation with mucositis grade in the overall cohort (r = 0.225, p = 0.005). When stratified by chemotherapy status, this correlation remained statistically significant in the group that received chemotherapy (r = 0.283, p = 0.015) but not in the group that did not receive chemotherapy. From week 3 onwards, no significant correlations were observed between either pre- or post-treatment RDW and mucositis grade across any subgroup. Visual trends suggest that the strength of this correlation diminished steadily in the weeks following week 2.

**Table 2:** Spearman Correlation Results for RDW (Pre and Post) With Mucositis Grade Note: Significant p-values (p < 0.05) are marked with an asterisk (*). Week 1 was treated as the baseline, and week 8 was excluded due to insufficient data (five patients). RDW: red cell distribution width

Week	Group	RDW_Pre_Correlation	RDW_Pre_P_Value	RDW_Post_Correlation	RDW_Post_P_Value	N_Observations
W2	Overall	0.062	0.443	0.225	0.005*	153
	Chemo (Y)	0.161	0.173	0.283	0.015*	73
	Chemo (N)	-0.055	0.627	0.172	0.127	80
W3	Overall	-0.026	0.753	0.12	0.14	153
	Chemo (Y)	0.151	0.203	0.113	0.343	73
	Chemo (N)	-0.207	0.066	0.1	0.377	80
W4	Overall	-0.001	0.987	-0.014	0.867	150
	Chemo (Y)	-0.049	0.683	-0.085	0.482	71
	Chemo (N)	0.041	0.718	0.061	0.596	79
W5	Overall	-0.006	0.937	-0.032	0.697	151
	Chemo (Y)	-0.003	0.981	-0.066	0.584	71
	Chemo (N)	-0.01	0.929	-0.082	0.472	80
W6	Overall	0.008	0.922	0.066	0.426	148
	Chemo (Y)	0.051	0.682	-0.006	0.959	68
	Chemo (N)	-0.026	0.821	0.136	0.23	80
W7	Overall	-0.012	0.886	-0.014	0.868	138
	Chemo (Y)	0.068	0.589	0.158	0.21	65
	Chemo (N)	-0.09	0.449	-0.131	0.268	73

Correlations between RDW and weight loss

In parallel, the study assessed the correlation between RDW and treatment-related weight loss (Table 3). Significant positive correlations emerged for post-treatment RDW during week 6 (r = 0.179, p = 0.027) and week 7 (r = 0.185, p = 0.025) within the overall cohort. Further subgroup analysis revealed that these associations were predominantly driven by the group that did not receive chemotherapy, where week 6 and week 7 correlations reached statistical significance (r = 0.225, p = 0.045 and r = 0.224, p = 0.049, respectively). No statistically significant correlations were observed in the group that received chemotherapy during any week. These findings suggest that post-treatment RDW may serve as a more relevant indicator of weight loss risk in patients not undergoing chemotherapy.

**Table 3:** Spearman Correlation Results for RDW (Pre and Post) With Weight Loss Note: Significant p-values (p < 0.05) are marked with an asterisk (*). Week 1 was treated as the baseline, and week 8 was excluded due to insufficient data (five patients). RDW: red cell distribution width

Week	Group	RDW_Pre_Correlation	RDW_Pre_P_Value	RDW_Post_Correlation	RDW_Post_P_Value	N_Observations
W2	Overall	0.037	0.651	0.117	0.149	153
	Chemo (Y)	0.087	0.464	0.104	0.383	73
	Chemo (N)	-0.007	0.949	0.121	0.287	80
W3	Overall	0.029	0.719	0.131	0.106	153
	Chemo (Y)	0.096	0.419	0.095	0.423	73
	Chemo (N)	-0.033	0.773	0.131	0.247	80
W4	Overall	-0.006	0.942	0.136	0.094	153
	Chemo (Y)	0.000	0.997	0.094	0.429	73
	Chemo (N)	-0.009	0.937	0.165	0.144	80
W5	Overall	-0.015	0.853	0.085	0.301	151
	Chemo (Y)	0.021	0.859	0.067	0.581	71
	Chemo (N)	-0.045	0.691	0.108	0.340	80
W6	Overall	0.000	0.998	0.179	0.027*	152
	Chemo (Y)	0.017	0.886	0.103	0.389	72
	Chemo (N)	-0.030	0.790	0.225	0.045*	80
W7	Overall	0.095	0.253	0.185	0.025*	148
	Chemo (Y)	0.095	0.432	0.089	0.462	70
	Chemo (N)	0.081	0.480	0.224	0.049*	78

RDW delta as a marker for weight loss

Beyond absolute RDW values, the RDW delta (defined as the difference between post-treatment and pre-treatment RDW) was evaluated for its association with weight loss. Notably, RDW delta demonstrated consistent and statistically significant positive correlations with weight loss in the overall cohort across all weeks analyzed. Specifically, significant correlations were observed from week 2 through week 7, with the strongest association found at week 6 (r = 0.253, p = 0.002), followed by week 4 (r = 0.198, p = 0.014) and week 7 (r = 0.195, p = 0.018). Importantly, subgroup analysis showed that the group that did not receive chemotherapy exhibited significant correlations at week 6 (r = 0.302, p = 0.007) and week 7 (r = 0.245, p = 0.031), while the group that received chemotherapy did not demonstrate any statistically significant findings across the same period.

RDW delta and mucositis grade

Contrary to its association with weight loss, RDW delta did not show a significant or consistent relationship with mucositis grade (Table 4). Across all weeks and treatment groups, correlation coefficients remained low, and none reached statistical significance. While some marginal trends were observed in certain weeks (e.g., week 3 in the group that did not receive chemotherapy: r = 0.200, p = 0.075), these did not pass the p < 0.05 threshold, indicating that RDW delta lacks associative utility for mucositis severity in this dataset.

**Table 4:** Spearman Correlation Results for RDW Delta With Mucositis Grade Note: Week 1 was treated as the baseline, and week 8 was excluded due to insufficient data (five patients). RDW: red cell distribution width

Week	Group	RDW_Delta_Correlation	P_Value	N_Observations
W2	Overall	0.015	0.065	153
	Chemo (Y)	0.161	0.175	73
	Chemo (N)	0.119	0.295	80
W3	Overall	0.082	0.314	153
	Chemo (Y)	-0.052	0.661	73
	Chemo (N)	0.200	0.075	80
W4	Overall	-0.033	0.691	150
	Chemo (Y)	-0.062	0.610	71
	Chemo (N)	0.004	0.975	79
W5	Overall	-0.031	0.710	151
	Chemo (Y)	0.050	0.677	71
	Chemo (N)	-0.056	0.624	80
W6	Overall	0.068	0.414	148
	Chemo (Y)	-0.043	0.729	68
	Chemo (N)	0.190	0.092	80
W7	Overall	-0.006	0.943	138
	Chemo (Y)	0.090	0.478	65
	Chemo (N)	-0.049	0.679	73

Association with adverse outcomes

To assess the associative value of RDW metrics, logistic regression analyses were conducted with two binary outcomes: severe weight loss (>5% and 10% from baseline) and severe mucositis (grade ≥3). The analysis revealed that among the three RDW measures - pre-treatment, post-treatment, and delta-only - RDW delta was statistically significantly associated with severe weight loss >10%. Specifically, RDW delta yielded an OR of 1.13 (95% CI: 1.01-1.27, p = 0.032), with an AUC of 0.60, indicating modest discriminative ability. Neither pre-treatment nor post-treatment RDW was significantly associated with weight loss or mucositis. Similarly, all RDW metrics failed to show a significant association with severe mucositis in the overall cohort, with p-values exceeding 0.7 and AUCs remaining below 0.52.

For overall severe weight loss >10%, RDW delta is a statistically significant marker associated with this outcome (OR 1.13, 95% CI: 1.01-1.27, p = 0.032), with an AUC of 0.60, indicating modest discriminative ability. Pre-treatment RDW and post-treatment RDW were not significantly associated with overall severe weight loss or mucositis. For severe mucositis, none of the RDW measures reached statistical significance in this cohort.

ROC curve analysis

**Figure 1:**
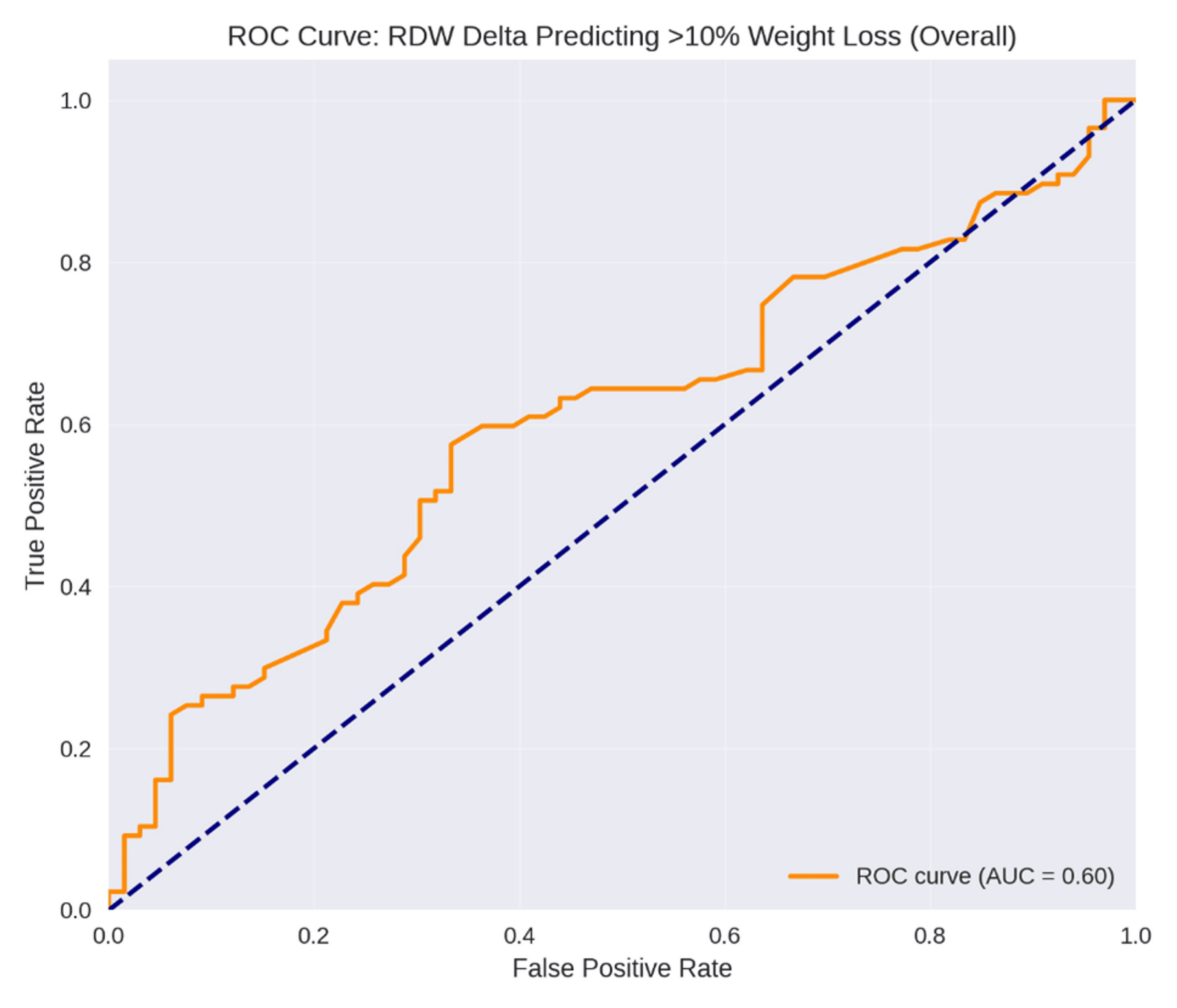
ROC Curve Analysis: RDW Delta as a Predictor of >10% Weight Loss RDW: red cell distribution width; ROC: receiver operating characteristic; AUC: area under the curve

ROC analysis further validated these findings. As shown in Figure 1, the ROC curve for the association of RDW delta with >10% weight loss yielded an AUC of 0.60, consistent with the logistic regression analysis. This finding reinforces the modest associative value of RDW delta for identifying patients at risk of clinically significant weight loss during treatment.

Week-by-week analysis

A more granular, weekly logistic regression analysis was performed to examine the temporal evolution of RDW metrics in their association with severe weight loss and mucositis. Notably, RDW delta consistently showed a significant association with weight loss exceeding 10% at multiple time points. At week 2, RDW delta was significantly associated with >10% weight loss (OR = 1.18, 95% CI: 1.04-1.34, p = 0.010, AUC = 0.656). This significant association persisted at week 3 (OR = 1.18, p = 0.007 AUC = 0.651), week 4 (OR = 1.18, p = 0.006, AUC = 0.634), and week 6 (OR = 1.15, p = 0.016, AUC = 0.623). These results suggest that RDW delta serves as a temporally stable biomarker for weight loss risk, with the most reliable associations occurring in the early to mid-phase of radiotherapy. In contrast, none of the RDW metrics reached statistical significance in their association with mucositis at any week. Although several models demonstrated AUC values in the range of 0.55 to 0.65, the lack of statistical significance and overall modest performance limit the clinical utility of RDW in this context.

Weekly ROC curve

**Figure 2:**
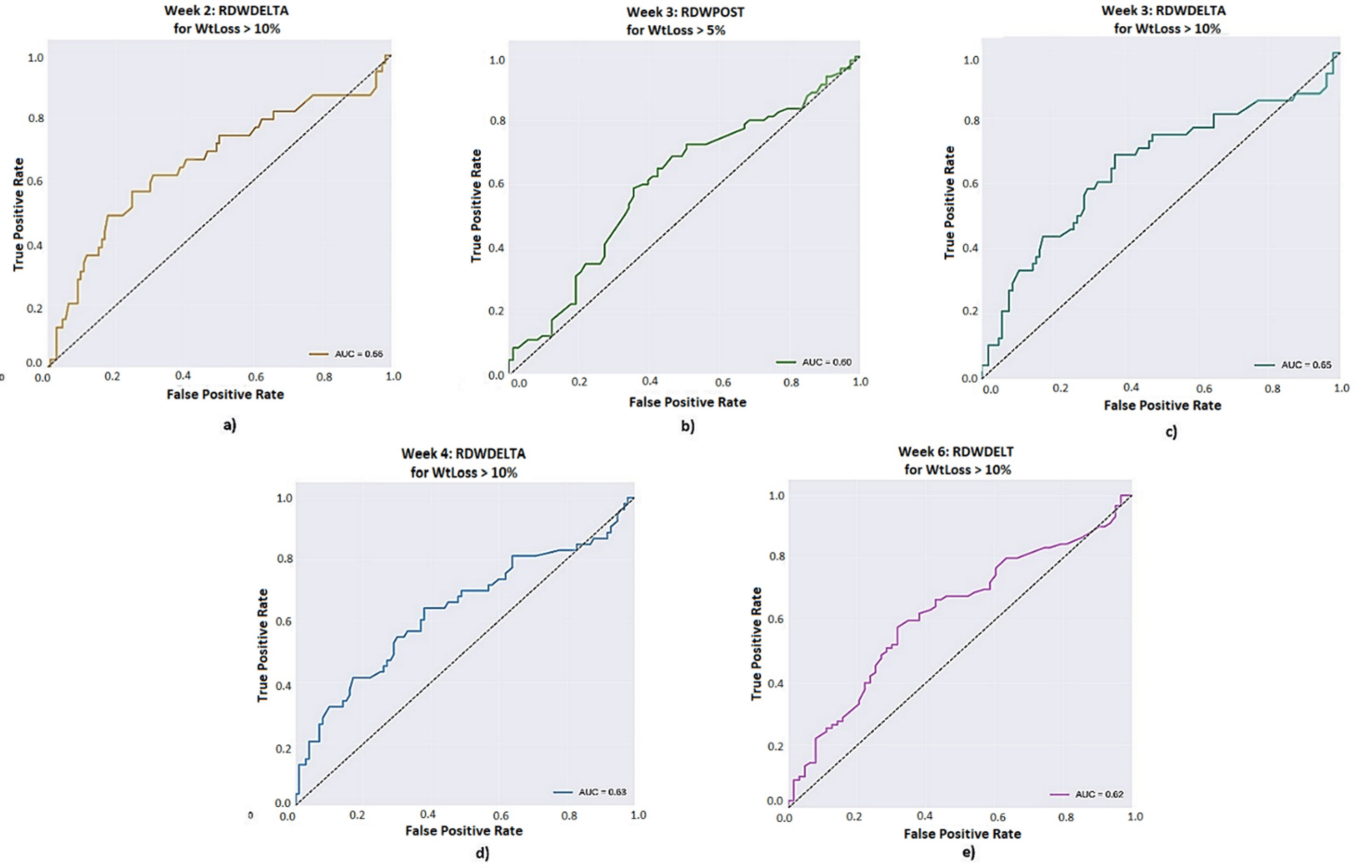
Weekly ROC Curve Analysis: RDW Delta as a Predictor of Weight Loss Across Radiotherapy Weeks The figure displays ROC curves illustrating the performance of RDW metrics in relation to significant weight loss. Each subplot illustrates the association between the RDW metric and the percentage of weight loss calculated for that specific week relative to the patient's baseline weight. The area under the curve (AUC) is provided for each model. RDW: red cell distribution width; ROC: receiver operating characteristic

Figure 2 visually summarizes the associative performance of RDW delta across weeks 2 to 6. The figure highlights that week 2 and week 3 yielded the highest AUC values (0.656 and 0.651, respectively), with consistent ORs above 1.0 for all significant associations. Although a slight decline in AUC was observed in later weeks, the trend remained directionally stable throughout the course of treatment. These findings confirm the early onset and sustained relevance of RDW delta as a marker for weight loss risk during radiotherapy.

Taken together, the results highlight RDW delta as the most promising and consistent RDW-related marker for predicting severe weight loss, particularly when using the >10% threshold. This association is most evident in the non-chemotherapy group and during the early weeks of radiotherapy. Conversely, none of the RDW metrics, including delta, showed meaningful or consistent predictive ability for mucositis grade across the study period.

## Discussion

The present study evaluated the utility of RDW and its temporal changes during radiotherapy as predictive markers for two critical adverse effects - weight loss and mucositis - in patients with head and neck cancer. Through comprehensive weekly analyses and logistic regression modelling, we identified RDW delta (change from pre- to post-treatment) as a consistent and modest predictor of severe weight loss, especially in patients not receiving concurrent chemotherapy. These findings align with emerging literature that recognizes RDW not just as a hematological parameter but also as a prognostic marker reflecting systemic inflammation and nutritional status.

Comparison with prior research on RDW and inflammation in cancer

Our findings are strongly supported by a meta-analysis conducted by Nocini et al. (2023), which systematically reviewed the prognostic significance of RDW in laryngeal cancer. They concluded that elevated RDW was significantly associated with poorer overall survival and disease-free survival, suggesting a relationship between RDW and tumor-driven systemic inflammation [2]. While our study focused on treatment-related outcomes rather than survival, the parallel in findings strengthens the argument that RDW may reflect an underlying inflammatory or catabolic milieu contributing to both cachexia and treatment intolerance.

Similarly, a retrospective study by Andrade et al. (2016) on Hodgkin lymphoma patients found that RDW >16.6% was independently associated with worse performance status, advanced stage, and poorer progression-free survival, suggesting that RDW captures an important dimension of disease severity and host response [7]. These parallels support the robustness of our conclusion that RDW delta, not absolute RDW values, offers the most dynamic insight into the evolving systemic state during cancer therapy.

RDW and weight loss: mechanistic insights

The pathophysiological rationale behind RDW’s association with weight loss may be multifactorial. RDW reflects anisocytosis, which increases in response to nutritional deficiencies (e.g., iron, folate, B12), systemic inflammation, and bone marrow dysfunction - all of which are prevalent in cancer patients undergoing radiotherapy. In our study, the strongest correlations between RDW delta and weight loss emerged from week 2 onwards, peaking at week 6. This trajectory mirrors known patterns of radiotherapy-induced catabolism, where mucosal injury, decreased intake, and metabolic stress converge to accelerate body mass depletion.

Our temporal findings are corroborated by Prystupa (2023), who demonstrated that RDW was an independent predictor of survival in patients with metastatic ovarian cancer, a group similarly vulnerable to chemotherapy-induced cachexia [8]. Likewise, Bento et al. (2016) reported that RDW >14.05% was associated with significantly higher mortality in diffuse large B-cell lymphoma (DLBCL), particularly due to comorbidities and inflammatory burden rather than treatment resistance, reinforcing the concept that RDW can reflect underlying systemic deterioration [9].

RDW and mucositis

In contrast to its association with weight loss, RDW failed to show a significant association with mucositis severity in our cohort. This divergence is noteworthy and highlights the complex etiology of mucositis, which is localized, dose-dependent, and influenced by genetic predispositions and oral microbiota, rather than by systemic inflammation alone.

This lack of predictive value is also consistent with observations from the broader literature. Although RDW has been implicated as a surrogate for systemic inflammation, it does not adequately capture site-specific tissue damage. A similar absence of predictive capacity was noted in the study by Afshar et al. (2019), where RDW failed to correlate with chemotherapy response in metastatic penile cancer, although it did correlate with overall survival [10]. These findings reinforce the interpretation that RDW reflects systemic health but may not serve as a biomarker for localized toxicities such as mucositis.

Temporal value of RDW delta: a biomarker in motion

Our study emphasizes RDW delta - the week-by-week change in RDW - as more informative than static measurements. This aligns with findings from Muhlestein et al. (2014), who demonstrated that RDW delta during hospitalization independently predicted mortality and length of stay in heart failure patients, even after controlling for baseline RDW [11]. Our data similarly showed that baseline RDW was not predictive, while RDW delta beginning week 2 consistently anticipated >10% weight loss with modest AUC values (~0.63-0.66).

The temporal advantage of RDW delta was also highlighted in a study by Henry et al. (2020), where elevated RDW at admission and its increase during hospitalization significantly correlated with COVID-19 severity and organ failure risk [12]. This consistency across disease domains underscores the emerging value of RDW delta as a dynamic systemic stress indicator.

Clinical implications and future directions

Taken together, these results suggest that RDW delta could be incorporated into routine toxicity monitoring for patients receiving radiotherapy. Its utility may be particularly valuable in resource-constrained settings, where advanced nutritional and inflammatory biomarkers (e.g., CRP, IL-6, albumin turnover) are not readily available.

Nevertheless, our study also highlights limitations in RDW’s association, with AUC values remaining under 0.66. This modest performance implies that while RDW delta may help identify at-risk patients early, it should not replace established assessment tools such as Patient-Generated Subjective Global Assessment (PG-SGA) or weekly weight tracking. Instead, RDW may serve as an adjunctive, low-cost red flag to trigger nutritional interventions or closer monitoring. It is also important to note that monitoring RDW delta may require more frequent blood draws than simple weekly weight tracking, making it a more invasive monitoring tool.

Future studies should explore whether combining RDW with inflammatory cytokines or nutritional indices could enhance predictive accuracy. For example, Cavusoglu et al. (2010) demonstrated that RDW retained independent prognostic value for mortality in cardiac patients even after adjusting for hematocrit and inflammatory comorbidities [13]. A similar multivariate approach could be adapted in the oncology setting to stratify nutritional risk more precisely.

Additionally, there is a growing call to investigate the molecular drivers of RDW elevation during radiotherapy. Inflammatory cytokines such as tumor necrosis factor-alpha (TNF-α) and interleukin-6 (IL-6), which inhibit erythropoiesis and increase red cell size variability, could mechanistically link treatment stress to RDW dynamics. As RDW is not causative but reflective, dissecting its underlying pathways may open doors to more targeted biomarkers and therapeutic interventions.

Strengths and limitations

A major strength of our study lies in its retrospective, week-by-week analysis across a relatively large cohort of head and neck cancer patients receiving standard-of-care treatment. The weekly granularity allowed for temporal mapping of RDW’s evolution, offering valuable clinical insights not captured in static cross-sectional designs.

However, limitations include attrition in later weeks, particularly week 7, and the exclusion of week 8 due to insufficient sample size. Additionally, the study was not designed to examine survival outcomes or quality-of-life indices, which would further contextualize RDW’s clinical relevance. Finally, external validation across different cancer types and treatment protocols is necessary to establish the generalizability of RDW delta as a toxicity biomarker.

Future directions

Future research should explore combining RDW with other hematologic ratios like neutrophil-to-lymphocyte ratio (NLR), platelet-to-lymphocyte ratio (PLR), or even systemic inflammation indices. Additionally, expanding studies into prospective, multi-institutional trials with a larger patient base would help validate RDW as a prognostic or predictive biomarker. The integration of machine learning techniques using multifactorial inputs, including RDW, inflammatory markers, treatment regimens, and demographic characteristics, might yield personalized models with superior predictive accuracy.

## Conclusions

This study highlights the clinical relevance of RDW, particularly RDW delta, as a marker associated with weight loss in patients undergoing radiotherapy for head and neck cancers. Among the hematological parameters assessed, RDW delta consistently demonstrated statistically significant correlations with weight loss from the second to seventh week of treatment, suggesting its potential as an early indicator of nutritional decline. These findings align with emerging evidence linking elevated RDW to systemic inflammation and cancer-related cachexia. In contrast, RDW showed minimal and inconsistent association with mucositis severity, achieving statistical significance only in the second week. This suggests that RDW may not adequately reflect localized mucosal injury or the complex pathophysiology underlying radiotherapy-induced mucositis. The utility of RDW, a routinely available and cost-effective laboratory parameter, offers a practical tool for early risk stratification and proactive nutritional intervention during cancer treatment. However, due to its modest predictive power and lack of specificity, RDW should be interpreted in conjunction with other clinical and inflammatory markers.

## References

[1] Eoh KJ, Lee TK, Nam EJ, Kim SW, Kim YT (2023). Clinical relevance of red blood cell distribution width (RDW) in endometrial cancer: a retrospective single-center experience from Korea. Cancers (Basel).

[2] Nocini R, Sanchis-Gomar F, Lippi G, Mattiuzzi C (2023). Red blood cell distribution width (RDW) is a significant predictor of survival in laryngeal cancer patients: systematic literature review and meta-analysis. J Med Biochem.

[3] Song Y, Huang Z, Kang Y (2018). Clinical usefulness and prognostic value of red cell distribution width in colorectal cancer. Biomed Res Int.

[4] Dang C, Wang M, Qin T, Qin R (2022). Clinical importance of preoperative red-cell volume distribution width as a prognostic marker in patients undergoing radical surgery for pancreatic cancer. Surg Today.

[5] Yüksel C, Erşen O, Culcu S, Bakırarar B, Unal AE, Demirci S (2021). Prognostic role of red distribution width (RDW) value in gastric cancer. J Coll Physicians Surg Pak.

[6] Ge W, Xie J, Chang L (2018). Elevated red blood cell distribution width predicts poor prognosis in patients with oral squamous cell carcinoma. Cancer Manag Res.

[7] Andrade BL, Robredo B, Sartori F (2016). Red cell distribution width (RDW) at diagnosis is associated to advanced stage, worse response and poor prognosis in Hodgkin lymphoma. Blood.

[8] Prystupa T, Obukhova NP (2023). Prognostic marker of red cell distribution width (RDW) correlates with survival outcomes in metastatic ovarian cancer patients. Am J Biomed.

[9] Bento L, Sarmentero J, Ortuño A (2016). Red cell distribution width (RDW) at diagnosis of diffuse large B-cell lymphoma (DLBCL) is associated to higher mortality and worse overall survival. Blood.

[10] Afshar M, Patel R, English L (2019). MP73-14 red cell distribution width (RDW) as a predictor of survival outcomes with palliative chemotherapy for metastatic penile cancer. J Urol.

[11] Muhlestein JB, Lappe DL, Anderson JL (2016). Both initial red cell distribution width (RDW) and change in RDW during heart failure hospitalization are associated with length of hospital stay and 30-day outcomes. Int J Lab Hematol.

[12] Henry BM, Benoit JL, Benoit S (2020). Red blood cell distribution width (RDW) predicts COVID-19 severity: a prospective, observational study from the Cincinnati SARS-CoV-2 emergency department cohort. Diagnostics (Basel).

[13] Cavusoglu E, Chopra V, Gupta A, Battala VR, Poludasu S, Eng C, Marmur JD (2010). Relation between red blood cell distribution width (RDW) and all-cause mortality at two years in an unselected population referred for coronary angiography. Int J Cardiol.

